# Hepatocellular Carcinoma With Tumor Thrombus Extension Into the Right Atrium of the Heart

**DOI:** 10.7759/cureus.46582

**Published:** 2023-10-06

**Authors:** Nova B Mebane, Rosemary E Wells, Manzoor Bevinal

**Affiliations:** 1 Internal Medicine, Corpus Christi Medical Center, Corpus Christi, USA; 2 Internal Medicine, Texas College of Osteopathic Medicine, Fort Worth, USA

**Keywords:** inferior vena cava tumor thrombus, transarterial chemoembolization (tace), atezolizumab plus bevacizumab, right atrium tumor thrombus, advanced hepatocellular carcinoma

## Abstract

Hepatocellular carcinoma (HCC) is the most common primary liver tumor. Most patients present to the hospital with systemic symptoms and typically have a history of liver disease. This case study involves a male in his 60s who presented to our hospital facility with a chief complaint of abdominal pain. He reported no history of liver disease but was diagnosed with HCC stage IVB during this hospitalization. Upon further imaging, a tumor thrombus was found invading the inferior vena cava with an extension into the right atrium. Our primary learning point in this article is to emphasize the importance of prompt surveillance for primary care physicians with patients who are at increased risk for HCC. Additionally, we discuss specific management aimed toward patients diagnosed with a tumor thrombus extending into the right atrium. This management includes dual immunotherapy (atezolizumab and bevacizumab) and transarterial chemotherapy embolization (TACE).

## Introduction

Hepatocellular carcinoma (HCC) is the most common primary liver tumor and is usually associated with chronic liver disease. The inflammatory reactions associated with chronic liver disease can create the setting for mutations resulting in HCC. Patients can present with nausea, vomiting, fatigue, weight loss, abdominal pain, jaundice, and other systemic symptoms. HCC is more common in Africa and Southeast Asia, due to the higher rate of hepatitis in those areas. HCC is more commonly diagnosed in males [1]. Other risk factors for HCC include excessive alcohol consumption, obesity, metabolic syndrome, viral hepatitis, smoking, cirrhosis, nonalcoholic steatohepatitis, and inherited disorders, such as alpha-1 antitrypsin deficiency, glycogen storage diseases, hemochromatosis, congenital biliary atresia, and Wilson’s disease [2].

The staging of HCC determines which treatment will be used. This cancer has several different growth patterns, including nodular, massive, and diffuse. Nodular HCC has a capsule and can include more than one nodule of growth. Massive HCC is a bigger growth that is not encapsulated. Diffuse HCC has many small areas of tumor growth throughout the liver. These various growth patterns are strongly considered for the patient’s treatment. Unfortunately, HCC has a poor prognosis even with treatment. This is partly due to the asymptomatic nature of HCC in the early stages. Thus, HCC is often discovered in the later stages of the disease.

According to the American Cancer Society (ACS), screening should only be performed on high-risk patients. These include patients with cirrhosis, hepatitis B infection, and hereditary hemochromatosis. Screening includes a liver ultrasound and/or alpha-fetoprotein (AFP) measurement every six months according to the American Association for the Study of Liver Disease (AASLD). Unfortunately, primary care surveillance rates for HCC have been 16.9% among American patients [3]. This surprisingly low percentage is likely due to multiple factors, including the education of healthcare providers and patients about this recommended surveillance [4].

Surgical interventions for HCC include liver resection and liver transplantation. Nonsurgical interventions/procedures include microwave ablation, percutaneous ablation, and trans-arterial chemoembolization. Many patients with diffuse HCC present with advanced disease in which surgical interventions are not indicated. Systemic treatment is often preferred, which includes immunotherapy and chemotherapy. Both systemic and liver-specific therapies may be considered, as seen in this case study with the utilization of trans-arterial chemoembolization (TACE).

HCC can be accompanied by various complications. Budd-Chiari syndrome can result from compression or invasion of the hepatic veins. HCC can spread vascularly; therefore, it is not uncommon for HCC to invade the portal system. However, instances of tumor extension into the heart are rare with a prevalence of 1-4% of patients diagnosed with HCC [5]. When these cases occur, they are usually discovered at autopsy rather than living patients. Cases of diffuse HCC with tumor extension to the right atrium after TACE treatment, as presented in this case study, tend to have a post-treatment survival range of two months to one year [6].

## Case presentation

The patient presented to our hospital in his 60s and was male. He had a past medical history of hypothyroidism, hypertension, gout, alcohol use, and a two-day recent diagnosis of liver cirrhosis and liver mass. He presented to our emergency department with a chief complaint of right upper quadrant abdominal pain. The pain was described as sharp and located under his right ribs and was present no matter what position he was in. He reported nausea, vomiting, bloating, intermittent diarrhea and constipation, and 20 lb weight loss in the last two weeks. He denied hematemesis, hematochezia, melena, fever, and chills.

His recent medical history included a primary care visit secondary to his severe abdominal pain in which they prescribed lactulose and recommended him to seek urgent care if the pain persists. He went to an outside facility emergency room two days prior to admission at our facility. The CT abdomen and pelvis without contrast obtained from this outside facility showed poorly visualized isodense and slightly hyperdense mass in the right lobe of the liver measuring at least 10x7x7.7 cm, cirrhosis, and splenomegaly. Additionally, he was diagnosed with an acute renal injury, received intravenous fluids, and was discharged home from the emergency department. The patient was advised to follow up with an oncologist, but the abdominal pain was too severe, and he decided to go to our hospital facility two days later. The patient was admitted to our hospital for abdominal pain secondary to liver mass as well as acute kidney injury.

Pertinent lab values from hospital admission include hemoglobin of 13.8 g/dl (rr=14-17 g/dl), hematocrit of 42.8% (rr=42-52%), platelet count of 139x10^3^/uL (rr=150-450x10^3^/uL), white blood cell count of 6.21x103/uL (rr=4.8-10.8x103/uL), blood urea nitrogen of 39 mg/dl (rr=6-20 mg/dl), creatinine of 1.94 mg/dl (rr=0.6-1.0 mg/dl), glomerular filtration rate of 38 ml/min (rr=49-113 ml/min), hemoglobin of A1c 5.4% (rr=4.5-6.2%), total bilirubin of 1 mg/dl (rr=0-1 mg/dl), aspartate transferase of 484 units/L (rr=15-37 units/L), alanine transferase of 58 units/L (rr=30-65 units/L), alkaline phosphatase of 149 units/L (rr=50-136 units/L), pro B-type natriuretic peptide of 949 Pg/Ml (rr=0-125 Pg/Ml), albumin of 3.3 g/dl (rr=3.4-5.0 g/dl), globulin of 5.3 g/dl (rr=1.5-3.8 g/dl), lipase of 206 units/L (rr=73-393 units/L), alfa fetoprotein (AFP) of 5,584 ng/ml (rr=0.0-8.4 ng/ml), thyroid-stimulating hormone of 4.44 miU/L (rr=0.42-5.47 miU/L), and uric acid of 6.9 mg/dl (rr=2.6-6.0 mg/dl). Additionally, the urinalysis was unremarkable, except for pH 5 (rr=5.5-7.0), the hepatitis panel was negative, and electrolytes (sodium, potassium, chloride, magnesium, calcium) remained within normal limits.

**Figure 1:**
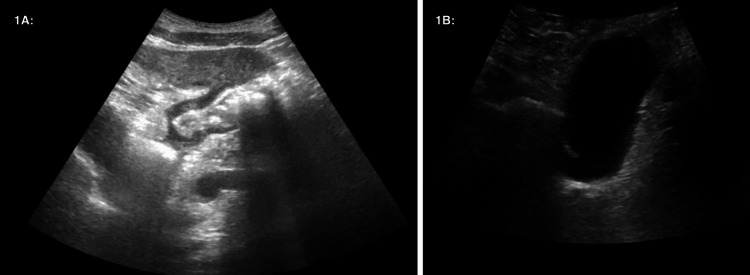
Right Upper Quadrant Ultrasound Performed at Hospital Admission. *1A:* There is mild hepatomegaly with heterogeneous poorly delineated infiltrating mass-like regions throughout the right hepatic lobe and a discrete 1.6 cm hypoechoic nodule in the left hepatic lobe. There is a discrete 1.6 cm hyperechoic nodule in the right hepatic lobe. Underlying neoplastic disease suspected. Recommend multiphase CT of the liver for further evaluation. *1B: *The distended gallbladder is sonolucent, with no gallbladder wall thickening, pericholecystic fluid, or stones. The common bile duct measures 4.8 mm.

**Figure 2:**
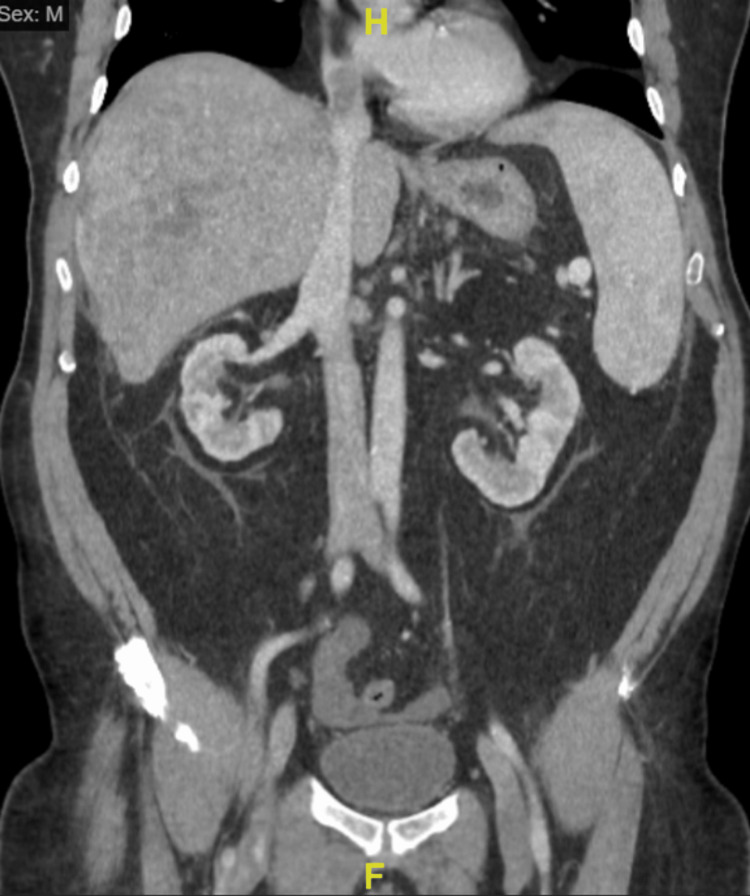
CT Abdomen and Pelvis Without Contrast Performed at Hospital Admission. Multiple hepatic lesions with findings concerning for tumor thrombus within the right hepatic and main portal veins with extension to the inferior vena cava and right atrium. Multiple hepatic lesions, the largest within the right hepatic lobe measuring 12.6x2.8x10.6 cm, exhibiting heterogeneous enhancement. In the setting of cirrhosis, this is concerning for multicentric hepatocellular carcinoma, and metastatic disease is difficult to exclude. Sequela of portal hypertension including splenomegaly, dilated main portal vein, and trace ascites. Colonic diverticula without evidence of diverticulitis. Mild prostatomegaly. Yellow "H" indicates the direction of head, while yellow "F" indicates the direction of feet.

Imaging studies were performed. A right upper quadrant abdominal ultrasound was performed, and findings were reported in Figure 1. CT abdomen and pelvis were performed, and findings were reported in Figure 2. Chest X-ray showed no acute cardiopulmonary findings. Retroperitoneal ultrasound showed no acute abnormality involving the kidneys with a mildly thickened bladder may be from chronic outlet obstruction or cystitis. The echocardiogram showed an ejection fraction of 65-69% with normal wall motion, systolic function, and valvular function. There was a pacer wire noted in the right ventricle; however, the patient did not have a history of pacemaker insertion, and this finding was later amended and re-interpreted as a right atrium tumor thrombus.

Procedures performed include esophagogastroduodenoscopy (EGD) and liver biopsy. The EGD showed no esophageal or gastric varices, but the presence of moderate portal hypertensive gastropathy. The liver biopsy showed cirrhosis with mild-to-moderate chronic inflammation and hepatocyte atypia consistent with dysplasia, but nondiagnostic of malignancy. Immunohistochemical studies of the liver biopsy show strong staining for arginase with nonspecific staining seen for glypican within one biopsy and negative staining in the second biopsy.

The patient’s clinical condition of abdominal pain improved with the ability to tolerate food. The patient was discharged with recommendations to follow up with his medical oncologist and primary care physician for further management of his diagnosis of hepatocellular cancer with vascular spread to the right atrium. He was given a short supply of opioid analgesics for breakthrough pain until he was able to establish an appointment with primary care and oncology providers.

After hospitalization, the patient immediately established care at a medical oncology clinic where he was diagnosed with hepatocellular carcinoma stage IVB TNM: cT3, cN1, and cM1. He began an immunotherapy regimen with atezolizumab and bevacizumab two weeks after hospitalization. Throughout his first course of immunotherapy, the patient had intermittent right upper quadrant abdominal pain and shortness of breath.

**Figure 3:**
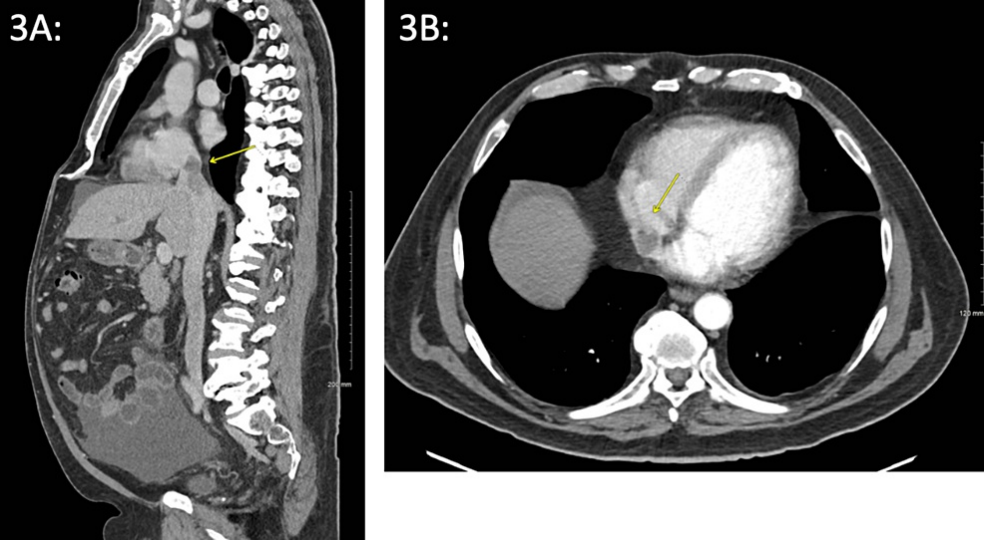
CT Abdomen and Pelvis with Contrast Performed After the First Cycle of Immunotherapy. *3A: *Sagittal view with a yellow arrow indicating the tumor thrombus invading the inferior vena cava. *3B: *Transverse view with a yellow arrow indicating the tumor thrombus invading the right atrium. Radiologist interpretation: A large area of diffuse hypoattenuation and scattered areas of enhancement nearly replacing the right hepatic lobe indicative of large hepatocellular carcinoma, as shown in previous imaging prior to immunotherapy. Tumor thrombus within the intrahepatic and suprahepatic inferior vena cava reaching the right atrium is still shown. Splenomegaly and moderate ascites suggest portal hypertension.

After finishing his third round of immunotherapy (two months after hospitalization), a CT chest and abdomen with contrast was performed, shown in Figure 3 with the radiologist’s interpretation of the study. At this time of evaluation, the medical oncologist noted an Eastern Cooperative Oncology Group (ECOG) performance status of 1, indicating that the patient is restricted in physically strenuous activity but ambulatory and able to carry out work of a light or sedentary nature.

In addition to immunotherapy, he was a candidate for TACE treatment. He underwent his first procedure approximately three months after his hospitalization with no acute complications. The angiography report displayed a large hypervascular right hepatic tumor consistent with HCC measuring approximately 12.5 cm in diameter. Successful partial embolization of the right hepatic tumor was performed with 100-300 um LC beads led with 150 mg of doxorubicin. The interventional radiologist's recommendation was to have stages of chemoembolization every three to four months due to the large size of the tumor. Complete embolization would be a high risk for abscess formation or other complications.

**Figure 4:**
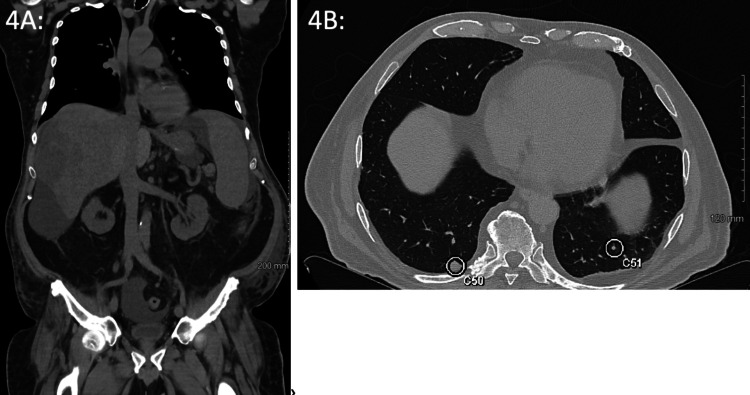
CT of the Chest, Abdomen, and Pelvis Without Contrast After TACE. *4A: *Cirrhosis of the liver with ascites and splenomegaly is present. Hypoenhancing mass is again seen in the posterior right lobe of the liver involving segments 8, 7, 6, and 5. The mass infiltrates liver parenchyma and is difficult to measure, but is larger in this study with a portion of the liver in the subdiaphragmatic region measuring 133 mm and previously measured 116 mm. Previous exams also showed a tumor thrombus in the right hepatic vein, but this cannot be seen in this study. The gallbladder and biliary system, pancreas, adrenal glands, kidneys, and retroperitoneum are normal, except for the borderline enlarged portacaval node, which measured 9 mm on the previous exam and now measures 10 mm. There is a definite lytic bone lesion in the midline of the S1 sacral segment showing no change from the prior exam, consistent with bone metastasis.* 4B:* This exam demonstrates 56 pulmonary nodules with previous initial CT imaging only showing four nodules. There is an interval increase in the pre-existing nodules. A pleural-based nodule on the posterior sulcus of the right lower lobe measures 11 mm in diameter and previously measured 6 mm (C50). Another pulmonary nodule is labeled as C51.

A restaging CT chest/abdomen/pelvis without contrast study was performed after his first chemo-embolization treatment, shown in Figure 4 with the radiologist’s interpretation of the study. This imaging was significant for detecting 56 pulmonary nodules in which only four nodules were detected 10 weeks earlier. He has completed his sixth cycle of atezolizumab and bevacizumab and will continue for three more cycles at which another restaging CT scan will be performed.

## Discussion

We admitted the patient with knowledge of a recently discovered liver mass at an outside facility, but the full extent of the tumor was not known until after further imaging. During the patient’s hospital course, we were also able to see the extension of the tumor into the right atrium and make a diagnosis of hepatocellular carcinoma stage IVB in a patient with no known history of liver disease. This diagnosis was made without the support of a positive liver biopsy. Even though a liver biopsy is the gold standard for diagnosing hepatocellular carcinoma, the negative predictive value is low [7]. Contrary to many other solid tumors, HCC does not require a liver biopsy for diagnosis. Diagnosis of HCC can be made due to multiple factors, including imaging evidence of liver tumor with spread to the right atrium, highly elevated AFP, and patient risk factors for HCC, such as excessive alcohol use and obesity.

AFP is the most used marker for hepatocellular carcinoma. The sensitivity for AFP test for malignancy is 58-68%, and the specificity is 80-94%, while noting that 30-40% of hepatocellular carcinoma patients have AFP within normal values [8]. Even though novel hepatocellular carcinoma biomarkers are still being researched, AFP is still the most utilized [9]. It is important to note that AFP serologic testing should not be performed as a screening modality, but more for tracking the progression of already established cancer.

While surgical resection is a treatment option to consider for HCC, many tumors are found at such an advanced stage that surgery is not feasible. Other exclusionary factors for surgical intervention include widespread involvement of the liver and postoperative complications. Other treatments for hepatocellular carcinoma must be considered and explored. TACE is one such treatment. The first large-scale study done by Jun et al. in 2014 compared the course of hepatocellular carcinoma in patients with and without tumor extension into the right atrium in stage IVB [10]. The results of this study showed that treatment may prolong survival, with TACE found to be the most commonly used and most effective. However, even with treatment, the median survival rate was 123 days [10]. There are two different TACE procedures. First is conventional transarterial chemo-ablation with lipiodol, and second is transarterial chemoablation with drug-eluting beads. TACE reduces blood supply to the tumor in an attempt to trigger tumor necrosis. However, collateral circulation may develop due to tumor upregulation of VEGF. Therefore, repeated sessions of TACE are necessary. These interventions are not curative but focus on palliative patient care by prolonging survival time [11].

It is debatable in the scientific literature that pulmonary metastasis is a complication after TACE in patients with HCC. Pulmonary complications of TACE include pulmonary oil embolism, interstitial pneumonitis, chemical pneumonitis, acute lung injury, lipoid pneumonia, acute eosinophilic and neutrophilic pneumonia, bilious pleuritis, pulmonary abscess, and pulmonary tumor embolism. It is presumed that a pulmonary tumor embolism can be the cause of lung metastasis after TACE. This case example supports this presumption that TACE could have aided with lung metastasis due to prior imaging evidence showing four lung nodules and post-TACE showing 56 nodules within the span of 10 weeks. More research will have to be conducted to establish a true connection.

As of the publication of this article, there is still no targeted therapy specifically against HCC with tumor thrombus extension into the right atrium; thus, we utilized medical guideline-directed treatment for infiltrative hepatocellular carcinoma. Systemic therapy with atezolizumab and bevacizumab was begun for our patient due to current scientific evidence of having better overall and progression-free survival than sorafenib [11]. In a phase three trial by Raoul et al. in 2019, atezolizumab-bevacizumab had an overall survival at 12 months of 67.2% (95% CI=61.3-73.1) as opposed to sorafenib of 54.6% (95% CI=45.2-64.0) [12]. Additionally, Australia has enacted this combination therapy of atezolizumab and bevacizumab, instead of the historical monotherapy with sorafenib based on the Imbravel50 trial from the New England Journal of Medicine in 2020 for treatment of HCC [13]. 

Unfortunately, this patient’s cancer was first detected in its final stage of metastasis. This case demonstrates the importance of prompt diagnosis and treatment for patients suspected of having HCC. A prior medical history review of this patient showed poor follow-up to annual wellness appointments partly due to generalized anxiety toward healthcare. It is important to encourage patients to have annual health visits with routine lab work performed and obtain a liver ultrasound when patients present with chronic abdominal pain and a history of alcohol use. Additionally, patients should be advised to discontinue modifiable risk factors for HCC, including smoking and alcohol use, and obtain hepatitis B and C virus immunization. Barriers to primary care access should be investigated to promote annual follow-up care for preventative medicine such as cancer screenings.

Since the publication of this article, our patient has survived eight months since his initial diagnosis of HCC stage IVB and five months after his first TACE procedure. He has had a total of two TACE procedures.

## Conclusions

This case study was a unique presentation of a patient with no established medical history of liver disease being diagnosed with hepatocellular cancer stage IVB with tumor thrombus extension into the right atrium. Diagnosis of HCC is very difficult to obtain in the early stages, and this case study supports the need for increased healthcare provider screening/consideration of liver disease with every yearly visit. This is especially true in patients with increased risk for HCC.

Currently, through our extensive literature review, there are no accredited treatment guidelines for patients diagnosed with HCC and tumor extension into the right atrium of the heart. Thus, our patient has been treated with guidelines based on hepatocellular cancer stage IVB. Palliative care with systemic immunotherapy (atezolizumab and bevacizumab) has been shown to extend patient survival. He also underwent transcatheter chemo-embolization treatment. Our patient is still living upon the completion of this publication.
